# Anticipation of guilt for everyday moral transgressions: The role of the anterior insula and the influence of interpersonal psychopathic traits

**DOI:** 10.1038/srep36273

**Published:** 2016-11-03

**Authors:** Ana Seara-Cardoso, Catherine L. Sebastian, Eamon McCrory, Lucy Foulkes, Marine Buon, Jonathan P. Roiser, Essi Viding

**Affiliations:** 1 Division of Psychology and Language Sciences, University College London, UK; 2 Neuropsychophysiology Lab, CiPsi, University of Minho, Portugal; 3 Department of Psychology, Royal Holloway, University of London, UK; 4 Institute of Cognitive Neuroscience, University College London, UK; 5 Epsylon Laboratory, Montpellier University, France

## Abstract

Psychopathy is a personality disorder characterised by atypical moral behaviour likely rooted in atypical affective/motivational processing, as opposed to an inability to judge the wrongness of an action. Guilt is a moral emotion believed to play a crucial role in adherence to moral and social norms, but the mechanisms by which guilt (or lack thereof) may influence behaviour in individuals with high levels of psychopathic traits are unclear. We measured neural responses during the anticipation of guilt about committing potential everyday moral transgressions, and tested the extent to which these varied with psychopathic traits. We found a significant interaction between the degree to which anticipated guilt was modulated in the anterior insula and interpersonal psychopathic traits: anterior insula modulation of anticipated guilt was weaker in individuals with higher levels of these traits. Data from a second sample confirmed that this pattern of findings was specific to the modulation of anticipated guilt and not related to the perceived wrongness of the transgression. These results suggest a central role for the anterior insula in coding the anticipation of guilt regarding potential moral transgressions and advance our understanding of the neurocognitive mechanisms that may underlie propensity to antisocial behaviour.

In the past decade there has been increasing interest in the neurocognitive processes that underlie moral cognition^1^. However, we still do not fully understand how these processes may contribute to both atypical and typical morality. Why, for example, do some of individuals routinely engage in irresponsible and immoral behaviour? And, more importantly, why do these individuals engage in this kind of behaviour in spite of apparently being capable of appropriate moral reasoning?

Individuals with high levels of psychopathic traits, which include blunted affect and a lack of empathy and guilt, have an increased risk of engaging in irresponsible and antisocial behaviours^2^. Yet, they do not appear to differ from individuals with low levels of these traits in relation to their moral judgment ability, i.e. the ability to judge whether an action is immoral or not^3^,^4^,^5^,^6^,^7^,^8^,^9^ (though see refs 10, 11 for exceptions). However, they do report less difficulty in making decisions when faced with moral dilemmas^6^,^7^,^9^ and present diminished neural responses in the amygdala and other regions typically associated with affective processing when they perform moral judgment tasks^4^,^5^,^8^,^12^,^13^. Atypical moral behaviour in these individuals seems to stem not from an inability to compute moral judgments, but rather from a disruption of the affective and motivational components of moral processing that may be important for adjusting one’s behaviour so as not to harm others^3^,^14^,^15^. In other words, individuals with high levels of psychopathic traits seem to *know* what is wrong, but do not *feel* it to be wrong; and therefore fail to inhibit actions that may harm others.

Previous neuroimaging studies on moral processing in psychopathy have relied on paradigms that involved judging actions from a third person perspective^5^,^13^ and judging highly hypothetical and often extreme moral dilemmas (e.g. killing one person to save the lives of many)^4^,^8^. It is still unclear whether individual differences in psychopathic traits are associated with atypical neural processing of first-person scenarios for more normative moral transgressions that could happen in everyday life, and whether atypical neural processing relates to emotional and motivational aspects of everyday moral behaviour. Recent work indicates that the adherence to moral and social norms largely depends on emotional processes, rather than on the ability to make moral judgements^16^,^17^. Guilt proneness (the predisposition to experience negative feelings about personal wrongdoings), in particular, has been found to consistently predict appropriate moral behaviour^18^ and to be negatively associated with levels of psychopathic traits^6^,^19^. Guilt can provide immediate and salient feedback on either executed or imagined behaviour^16^ and the anticipation of feelings of guilt about committing a transgression can thus work as a ‘powerful brake’ that curbs antisocial or immoral behaviour. Investigating the neural correlates involved in processing the anticipation of guilt about committing more ordinary everyday moral transgressions may provide important cues to the mechanisms that underlie propensity to antisocial behaviour.

To address these questions, we developed a novel fMRI task with guilt-eliciting everyday moral scenarios, and set out to test whether variance in psychopathic traits is associated with neural processing of anticipated guilt for personal, everyday moral transgressions. Inside the scanner, participants were instructed to imagine themselves in each scenario and to rate how guilty they would feel; this allowed us to identify regions that parametrically encoded the strength of anticipated feelings of guilt. Subsequently, we conducted a follow-up study where a second group of participants performed a variation of the everyday moral scenarios fMRI task in which participants were asked to assess the wrongness of the action presented. This was to test whether any pattern of processing related to psychopathic traits was linked with the anticipation of feelings of guilt specifically, as opposed to the assessment of the ‘wrongness’ of the transgression. As discussed above, although adherence to moral and social norms has been hypothesised to depend largely on subjective emotional responses, rather than the ability to tell right from wrong^16^,^17^,^20^,^21^; these two processes have not been explicitly disambiguated in previous neuroimaging studies investigating moral processing and psychopathic traits.

We predicted that the processing of everyday personal moral transgressions would elicit responses in brain regions that have consistently been associated with moral cognition, including temporo-parietal junction (TPJ), supramarginal gyrus (SMG), anterior insula (aINS), amygdala and ventromedial prefrontal cortex (vmPFC)^1^,^22^,^23^,^24^,^25^. We further predicted that modulation of trial-by-trial variation in feelings of anticipated guilt (but not wrongness) would be observed in the aINS. The aINS is thought to support interoception of subjective emotional states^26^,^27^, has been consistently identified in previous work examining the neural correlates of guilt^17^,^28^,^29^,^30^,^31^ and has been shown to vary in activation with the strength of overall emotional appraisals of moral situations^24^,^32^, pointing to an important role for this region in encoding aversive responses to moral transgressions. Finally, based on the hypothesis that atypical moral behaviour observed in individuals with high levels of psychopathic traits is rooted in motivational/affective impairments, we predicted that responses in regions encoding the anticipation of feelings of guilt (but not wrongness) about committing moral transgressions would be negatively associated with psychopathic traits.

## Results

### Task and questionnaire data

**Figure 1:**
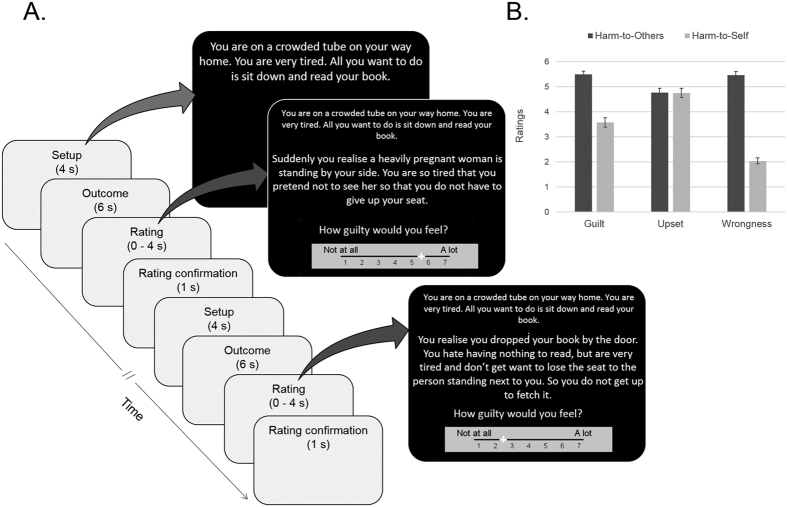
Everyday moral transgressions task. (**A**) Task timeline for two non-consecutive trials. Participants were presented with each scenario over three screens, representing each phase of the trial: 1) Presentation of the personal goal (‘Setup’; 4 s); 2) Presentation of the ending, i.e. harm-to-other or harm-to-self (‘Outcome’; 6 s); 3) Rating of guilt (in the Guilt task) or wrongness (in the Moral Judgment task) on a sliding scale (‘Rating’, 0–4 s); (**B**) Manipulation check. Ratings of ‘Guilt’ and ‘Upset’ (Guilt task) and ‘Wrongness’ (Moral Judgment task) on harm-to-other and harm-to-self trials. These scenarios elicited similar levels of negative emotional state (t_(27)_ = −0.02; p = 0.99), but differed in terms of levels of guilt (t_(27)_ = 8.96; p < 0.001). Moral judgments (i.e. wrongness judgments) ratings were significantly higher in harm-to-other scenarios compared with harm-to-self (t_(28)_ = 24.29; p < 0.001). Error bars represent standard error of the mean.

Our fMRI task (Fig. 1) comprised scripts of scenarios of potential everyday moral transgressions that typically evoke a sense of personal culpability. Scenarios were presented in the second person, as if the participant was the agent, and described personal goals achieved by causing harm either to another person or to oneself, thus representing a moral transgression or a morally neutral (but similarly unpleasant) situation, respectively. The latter acted as our control condition. Inside the scanner, participants (32 males recruited from the community) were instructed to imagine themselves in each scenario and to rate how guilty they would feel. After scanning, as a post-task manipulation check to ensure that both types of scenarios elicited different levels of guilt but similar levels of negative affect, participants were asked to read the scenarios for a second time and to rate how upset they would feel in each situation. As expected, ‘guilt’ ratings were significantly higher in scenarios depicting moral transgressions (harm-to-other scenarios) compared with control scenarios (harm-to-self scenarios) (t(27) = 8.96; p < 0.001; ‘harm-to-other’: M = 5.50, SD = 0.62; ‘harm-to-self’: M = 3.57, SD = 1.03). By contrast, both types of scenarios elicited similar levels of negative affect: ‘upset’ ratings were comparable between the conditions (t(27) = −0.02; p = 0.99; ‘harm-to-other’: M = 4.76, SD = 0.93; ‘harm-to-self’: M = 4.75, SD = 0.97).

Psychopathic traits were assessed using the Self-Report Psychopathy Scale Short-Form (SRP^33^), a well validated measure comprising affective (e.g. callousness), interpersonal (e.g. deceitfulness), lifestyle (e.g. impulsiveness) and antisocial behaviour facets of psychopathy. Interpersonal and lifestyle psychopathic traits were significantly negatively associated with ratings of anticipated guilt in moral transgressions scenarios (interpersonal: r = −0.53, p < 0.01; lifestyle: r = −0.43, p = 0.02; respectively; Table S1). There were no significant associations between psychopathic traits and ratings of guilt in control scenarios, nor with ratings of upset in either type of scenarios (Table S1).

### Neural processing of anticipated guilt for everyday moral transgressions

**Figure 2:**
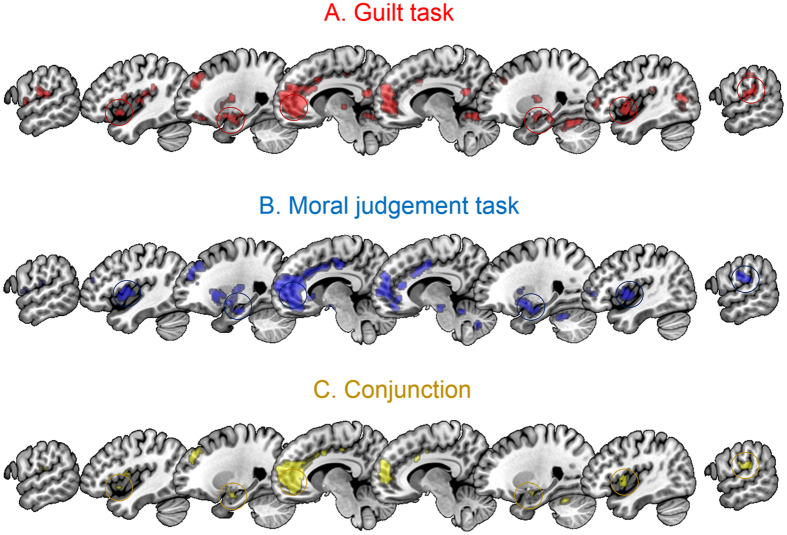
Neural correlates of everyday moral transgressions. Main effects (harm-to-other > harm-to-self) of the guilt task (**A**), the moral judgment task (**B**) and their conjunction (**C**). Significant activations across both tasks are present in the left anterior insula, left amygdala, ventromedial prefrontal cortex, right amygdala, right anterior insula and right supramarginal gyrus (highlighted, from left to right). Overlays are thresholded at p < 0.001 (uncorrected) and superimposed on the MNI 152 template brain provided in MRIcroGL^40^ for illustrative purposes.

First, to allow comparison with previous literature on the neural basis of moral cognition, we inspected brain regions that responded differentially to scenarios depicting moral transgressions (‘harm-to-other’) compared to control (‘harm-to-self’) scenarios (“main effect” model; see ‘Experimental Procedures’ and ‘Supplementary Experimental Procedures’ for full details, including statistical thresholds and region-of-interest (ROI) definition). Significant responses were observed in a network of brain regions that has consistently been associated with moral cognition^1^: aINS, SMG, vmPFC (all p < 0.05, whole-brain corrected (WBC)), and amygdala (p < 0.05, small-volume corrected (SVC)) (Table 1 and Fig. 2).

Critically, we inspected whether there were specific brain regions that parametrically encoded the strength of anticipated feelings of guilt about committing moral transgressions (“parametric modulation” model; see ‘Experimental Procedures’ and ‘Supplementary Experimental Procedures’ for full details, including statistical thresholds and region-of-interest (ROI) definition). We identified a positive parametric modulation of feelings of guilt elicited by moral transgressions in aINS at an uncorrected threshold of p < 0.001 (i.e. ratings of feelings of guilt correlated positively with BOLD response in this region on a trial-by-trial basis), although this did not survive correction for multiple comparisons ([x, y, z: −30 29 16], Z = 3.85, k = 4, p = 0.13, SVC).

### Associations between neural processing of anticipated guilt for everyday moral transgressions and psychopathic traits

**Figure 3:**
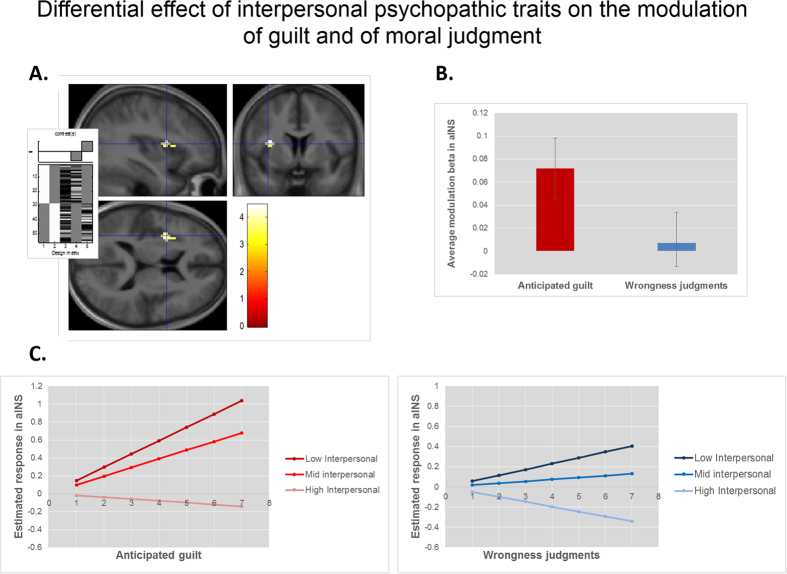
Modulation of feelings of guilt and wrongness judgments in anterior insula. (**A**) Cluster in the anterior insula where psychopathic interpersonal traits were associated with the modulation of activation by feelings of anticipated guilt, but not by wrongness judgments ([x, y, z: −36 5 13], k = 23, Z = 4.08, p > 0.05, SVC). The overlay is thresholded at p < 0.001 (uncorrected). Inset: second level SPM design matrix showing the contrast; columns (left to right) represent: 1) parametric modulation of anticipated guilt feelings (from Study 1); 2) parametric modulation of wrongness judgments (from Study 2); 3) age; 4) and 5) interpersonal psychopathic traits from Studies 1 and 2, respectively. (**B**) Bars depict the average regression slopes between trial-by-trial guilt/moral judgment ratings and BOLD response at the anterior insula peak voxel (from A); error bars represent standard errors of the mean. (**C**) Graphs illustrate the simulation of the modulation of activation by anticipated feelings of guilt (left) and judgments of wrongness (right) at the anterior insula for individuals with low, medium and high levels of psychopathic traits. For illustrative purposes, modulation betas (i.e. regression slopes between BOLD response and ratings) from the anterior insula peak voxel in A were extracted, participants were divided into tertiles of low, medium and high psychopathic interpersonal traits, and an average modulation beta value was computed for each tertile group and used to estimate the hypothetical BOLD response for each point of the rating scale.

We then examined whether variability in processing of anticipated guilt for everyday moral scenarios was associated with psychopathic traits by adding each facet of psychopathy separately as a regressor of interest in the parametric modulation model. We identified negative associations between the modulation of anticipated feelings of guilt and interpersonal psychopathic traits in the aINS, bilaterally (right: [x, y, z: 48 −1 −5], k = 11, Z = 3.84, p = 0.03, SVC; left: [x, y, z: −36 8 7]; k = 9, Z = 3.69, p = 0.04, SVC). To further understand the nature of these interactions, we extracted the parameter estimates (betas) from these clusters’ peaks. Note that these parameter estimates correspond to the regression slope linking the trial-by-trial BOLD response in aINS with the trial-by-trial variation in guilt ratings for each specific participant. For illustrative purposes only (Fig. 3), we divided participants into tertiles of low, medium and high psychopathic interpersonal traits, computed the average parameter estimate (i.e. the average regression slope) for each tertile group, and used these average parameter estimates to plot the increasing BOLD response in aINS along the guilt rating scale. Participants with lower levels of psychopathic interpersonal traits had steeper regression slopes; with relatively flat slopes for those with high levels of psychopathic interpersonal traits. That is, anticipated feelings of guilt increased linearly with BOLD response in aINS for those with low and medium levels of psychopathic interpersonal traits, but not for those with high levels of these traits.

For completeness, we examined whether variability in brain response evoked by guilt-eliciting moral transgression (vs. control) scenarios was associated with psychopathic traits (main effect model). Adding each facet of psychopathy separately as a regressor of interest in the main effect model identified positive associations between neural response and antisocial behaviour traits in the vmPFC ([x, y, z: −6 53 −11], k = 43; Z =  3.64, p = 0.05, SVC) and amygdala ([x, y, z: −21 −4 −14], k = 5, Z = 3.45, p = 0.02, SVC).

### Study 2: Dissociating neural processing of anticipated guilt from wrongness judgments evoked by moral transgressions

To confirm that this pattern of findings was specific to the modulation of anticipated guilt and not related to the perceived wrongness of the transgression, we conducted a follow-up study where we scanned a second group of 32 participants while performing a variation of the everyday moral scenarios fMRI task. In this task variation, participants judged the wrongness of the transgressions instead of rating anticipated guilt. All stimuli and task parameters were unchanged. This second group did not differ from the initial group in terms of means or variances in any psychopathic personality facet or total score. They did differ in age (t = 2.62, *p* = 0.01) and therefore age was added as a covariate in all comparison analyses (see ‘Experimental Procedures’ and ‘Table S2’ for full details).

#### Task and questionnaire data

Judgments of wrongness were significantly higher in moral transgression (harm-to-other) than in control (harm-to-self) scenarios (t(28)  =  24.29; p < 0.001). There were no significant associations between moral judgments in either type of scenarios and psychopathic traits (Table S1).

#### Neural processing of moral judgments about committing moral transgressions

As before, to allow comparison with previous literature on the neural basis of moral cognition and with our previous results, we inspected brain regions that responded differentially to scenarios depicting moral transgressions compared to control scenarios (“main effect” model; see ‘Experimental Procedures’ and ‘Supplementary Experimental Procedures’ for full details, including statistical thresholds and region-of-interest (ROI) definition). Analysis of the main effect model of this version of the task implicated a comparable set of regions as reported for the guilt task (clusters in vmPFC, SMG and aINS all p < 0.05, WBC; Table 1, Fig. 2; amygdala p < 0.05, SVC; Table 1). Conjunction analysis of the main-effects of the two tasks confirmed that the same set of regions was recruited during both tasks (Table 1; Fig. 2). The main effects did not differ significantly between the two tasks.

Analysis of the parametric modulation model (here incorporating wrongness judgments) revealed positive modulations in TPJ and SMG at an uncorrected threshold of p < 0.001 (i.e. wrongness ratings correlated positively with response in these regions), but these did not survive corrections for multiple comparisons (TPJ: [x, y, z: −60 −49 22], Z = 3.12, k = 1, *p* = 0.23, SVC; SMG: [x, y, z: −51 −25 22], Z = 3.43, k = 3, *p* = 0.10, SVC; [x, y, z: −57 −49 25], Z = 3.16, k = 2, *p* = 0.20, SVC). Such modulation was not evident in other regions, even at a more liberal uncorrected threshold of p < 0.005 (uncorrected). Conjunction analyses of the parametric modulators of the two tasks (i.e. wrongness judgments and feelings of guilt) yielded no significant results; in other words, in no region did the parametric relationships between BOLD responses and feelings of wrongness and guilt overlap. There were also no significant differential modulation effects between the two tasks.

#### Associations between neural processing of moral judgments for everyday moral transgressions and psychopathic traits

We found no significant effects of psychopathic traits in the moral judgment parametric modulation model, either at whole-brain or in ROIs. Critically, comparing the effects of interpersonal psychopathic traits on the two parametric modulation models (i.e., effect of interpersonal psychopathic traits on modulation of wrongness judgments compared to effect of interpersonal psychopathic traits on modulation of anticipated guilt) revealed a significant interaction in the aINS ([x, y, z: −36 5 13], k = 23, Z = 4.08, p = 0.05, SVC). The association of interpersonal psychopathic traits with the aINS modulation by feelings of guilt was significantly stronger than the equivalent association with the modulation by judgments of wrongness. To further understand the nature of this difference, we extracted and plotted the average modulation betas from this cluster’s peak, as described above. As shown in Fig. 3, the encoding of wrongness in aINS did not differ between groups with different levels of interpersonal psychopathic traits. Follow-up t-test analyses of the extracted modulation betas confirmed a significant positive parametric modulation effect in aINS of anticipated guilt (t = 2.72; p = 0.01, 1-tailed) but not of wrongness judgments (t = 0.35; 0 = 0.36, 1-tailed).

As before, for completeness, we examined whether variability in brain response evoked by judging the wrongness of harm-to-other (vs. harm-to-self) scenarios was associated with psychopathic traits (main effect model). No significant associations were found.

## Discussion

Processing potential personal everyday moral transgressions elicited responses in a distributed network of brain regions, consistent with that reported in previous neuroimaging studies investigating moral processing. These included: the SMG, reliably associated with processing self and other-related information; the amygdala and aINS, which have been strongly linked with affective processing; and the vmPFC, which has been hypothesised to integrate inputs from this distributed network to calculate the overall value of a given action^1^,^22^,^23^,^24^,^25^. Our conjunction analyses revealed that considering transgressions evoked responses in this network, irrespective of whether participants were anticipating feelings of guilt or were judging wrongness. Taken together with prior research, our results suggest that the same core circuit is engaged during moral processing, irrespective of the familiarity of the moral scenario (everyday vs. unusual) or of the nature of the processing (guilt antecipation vs. judgments of wrongness).

We also obtained a more fine-grained picture of the precise role that the different components of this circuit play in driving atypical moral behaviour, specifically identifying an interaction between the parametric modulation of aINS responses by anticipated feelings of guilt and levels of interpersonal psychopathic traits. The aINS parametrically encoded the strength of anticipated guilt, though not for those with higher levels of psychopathic interpersonal traits. It is thought that the aINS has a key role in the interoceptive awareness of subjective feelings^26^,^27^. The aINS has been identified in previous imaging studies where participants read guilt- eliciting sentences^29^ or stories^28^, recalled past guilt-eliciting situations^17^ or were given guilt-eliciting feedback^30^, indicating the importance of this region in processing feelings of guilt. Two recent studies have also shown that response in this region varies with the strength of the overall emotional appraisal of moral situations^24^,^32^. However, the nature of the tasks used in these previous studies precluded the inspection of whether the aINS response varied with the strength of feelings of guilt. Our results extend these previous findings, suggesting that it may play a critical role in signalling the strength of anticipated feelings of guilt about committing moral transgressions. At least in those with low-to-medium levels of interpersonal psychopathic traits.

Data from our second experiment confirmed that the negative association between the strength of anticipated guilt encoding in the aINS and individual differences in interpersonal psychopathic traits could not be explained by processing associated with wrongness judgments. Our results provide new evidence that psychopathic traits moderate the neural processing of guilt, rather than of moral judgments of wrongness, in line with the hypothesis that atypical moral behaviour in individuals with high levels of psychopathic traits is related to affective and motivational components of moral processing rather than in an inability to compute moral judgments^3^,^14^,^15^. Anticipation of guilt may influence behaviour, acting as a ‘‘moral brake’’ and decreasing the likelihood of those behaviours that will cause harm to others and guilt in ourselves. Insensitivity to anticipated guilt may help explain the lack of care for others’ well-being and difficulty in adhering to moral rules which are characteristic of individuals with high levels of psychopathic traits, despite apparent appropriate moral reasoning.

One limitation of our study is that the aINS encoding of the degree of anticipated guilt failed to reach significance after correction for multiple comparisons. It is possible that our study design was not optimally sensitive to detect this effect; a larger set of trials per task condition and a larger number of participants could represent improvements in this respect. However, a smaller effect for this relationship overall is perhaps unsurprising given that it showed modulation by individual differences in psychopathic traits, which may diluted the main effect. Likewise, it is possible that other individual differences effects explain why the encoding of wrongness judgments in TPJ/SMG did not survive stringent correction for multiple comparisons. These results show the importance of taking individual differences into account and show using individual differences such as psychopathic traits can shed light on the functioning of the typical system.

In conclusion, our results extend the understanding of the neurocognitive mechanisms involved in moral cognition and provide new links between these mechanisms and psychopathic traits. To our knowledge, this is the first study suggesting that the aINS plays a role in signalling anticipated feelings of guilt in response to potential everyday moral transgressions. We showed that the strength of this signalling varied negatively with individual differences in interpersonal psychopathic traits, consistent with the hypothesis that anticipation of guilt is attenuated in those with high levels of such traits. Importantly, we showed that this pattern of findings was specific to neural responses to anticipated feelings of guilt, and not linked to moral judgments. Our data align with models of atypical moral behaviour that propose that individuals with high levels of psychopathic traits may not lack the ability to compute moral judgments *per se*, but instead fail to generate the attendant negative affective states that usually inhibit harmful actions towards others, such as anticipated guilt.

## Methods

### Participants

After completing biographical screening questionnaires, two groups of 32 right-handed male participants with no reported history of neurological or psychiatric disorders were scanned. Following exclusions (see ‘Supplementary Experimental Procedures’ for full details), data from 28 participants in each group were analysed (mean age: 26.3 (Study1) and 23.0 (Study 2); age range 20–40 (Study 1) and 20–34 (Study 2)). Both studies were conducted in full accordance with the guidelines set by UCL Division of Psychology and Language Sciences Ethics Committee who provided ethical approval for this study. All participants provided written informed consent.

### Self-Report Psychopathy Scale Short-Form (SRP-SF; Paulhus *et al.* 2015)

The SRP-SF is a well-validated trait measure of psychopathy designed to measure psychopathic attributes in community samples. It is composed of four underlying facets indexing affective (e.g. “I never feel guilty over hurting others”), interpersonal (e.g. “I have pretended to be someone else in order to get something”), lifestyle (e.g. “I’ve often done something dangerous just for the thrill of it”) and antisocial (e.g. “I have broken into a building or vehicle in order to steal something or vandalize”) features of the psychopathic personality. The SRP-SF shows a clear latent structure, good construct validity^34^ and is strongly correlated with the Psychopathy Checklist-Revised^33^,^35^, the standard clinical interview measure for psychopathy. Items are scored on a 5-point Likert scale (from 1 “Disagree Strongly” to 5 “Agree Strongly”). In the present study, SRP-SF scores presented similar distributions to previousy reported distributions from larger samples of adults from the general population^7^,^33^,^36^,^37^ (please refer to ‘Supplementary Information’ for further details).

### Everyday moral transgressions task

We developed a novel, well-controlled task that presented scripts of realistic everyday moral scenarios for participants to read. Twenty-eight scenarios with two different endings were initially created for this task. These scenarios comprised descriptions of personal goals, each with two possible endings: causing harm to another person, or harm to oneself. These two endings thus represented either a moral transgression or a morally neutral (but still unpleasant) situation. The endings of the scenarios were matched in terms of: ratings of negative affect; participant perspective and agency; number of characters participating in the scenario; order of presentation of relevant information; and word number. Furthermore, all scenarios (both those containing harm to other and those containing harm to oneself) clearly indicated the intentionality of the protagonist and the consequences of the action. A two-phase pilot study was conducted to select the 15 best scenarios for the fMRI task in an independent sample. In the pilot study, 40 participants read the stories and were asked to imagine themselves as the protagonist, rate how guilty and upset they would feel, and how morally wrong the action in the story was. The scenarios chosen for the present study were those where the two endings were closely matched for negative affect (i.e. upset ratings on harm-to-other were not significantly different from upset ratings on harm-to-self), but where only the moral transgression ending elicited guilt and was judged as clearly morally wrong (i.e. mean ratings of harm-to-other scenarios were above the middle point of the guilt and of the wrongness judgment scales).

Prior to scanning, participants were familiarised with the task and instructions using practice stimuli. During scanning, participants were presented with 30 trials (15 scenarios with two endings each), and were instructed to imagine themselves in each situation and rate how guilty they would feel. Trials comprised three stages: 1) presentation of the personal goal (‘setup’; 4 s); 2) presentation of the ending, i.e. harm-to-other or harm-to-self to achieve the goal (‘outcome’; 6 s); and 3) rating of subjective guilt on a sliding scale (1 [‘Not at all’] to 7 [‘A lot’] after a prompt question ‘How guilty would you feel?’ (Study 1) or ‘How wrong would this be?’ (Study 2). Participants made their ratings using a keypad. Two keys moved the cursor (initially positioned in the centre of the scale) to the left or right, and a third key registered the answer. After registering their ratings, participants received visual confirmation of their answer for 1 s before the next trial started. Participants had a maximum of 4 s to make their ratings. If a rating was not made within that time, the trial was considered an error. Fifteen null trials, where the sentence ‘This is a small break, please keep still’ appeared on the screen for 10 s, were included. Trials were presented in a pseudorandom order to prevent more than two consecutive trials of the same type and more than one consecutive null trial. After scanning, as a manipulation check to ensure that both types of scenarios elicited similar levels of negative affect, participants were asked to read the scenarios for a second time and to rate how upset they would feel in each situation. To verify the scale integrity of the newly developed task, scale reliability analyses were conducted. All Cronbach’s alphas were good (guilt ratings in Study 1 (transgression scenarios: 0.81; control scenarios: 0.93), upset ratings in Study 1 (transgression: 0.89; control scenarios: 0.88; wrongness ratings in Study 2 (transgression scenarios: 0.81; control scenarios: 0.82)), suggesting good internal consistency (please refer to ‘Supplementary Information’ for descriptions of all scenarios).

### fMRI Data Acquisition and Analyses

A Siemens Avanto 1.5T MRI scanner at the Birkbeck-UCL Centre for Neuroimaging with a 32-channel head coil was used to acquire a 5.5 min 3D T1-weighted anatomical scan and multislice T2*-weighted echo planar images (EPIs) with BOLD contrast. The EPI sequence was based on^38^ (see ‘Supplementary Experimental Procedures’ for acquisition parameters, preprocessing pipeline and procedures for removing data corrupted by participant motion). Stimuli were presented using Cogent, running in Matlab 2011b (http://mathworks.com). Data were analysed in statistical parametric mapping (SPM).

For each version of the task, two first-level models were estimated for each participant: 1) to identify brain regions that responded differentially to the overall processing of moral transgressions (harm-to-self vs. harm to-other scenarios); 2) to identify regions that parametrically encoded the strength of feelings of guilt or wrongness judgments elicited by the moral transgressions. In the first models (‘main effect’), regressors corresponding to each condition were defined by the onset of the ‘outcome’ stage of the trial and lasted until the response (durations thus varied across trials from 6–10 s). This was to account for the fact that guilt- or wrongness-related processes may commence from the moment participants read the outcome right up until the point that ratings were given. Regressors were created by convolution of these boxcars with a canonical hemodynamic response function. Other events modelled in the analysis included: goal presentation (pooled across all scenarios); null trials; and errors (defined by non-response). Six realignment parameters were modelled as parameters of no interest in both analyses. The second models (‘parametric modulation’) were specified as the first model, but included guilt or wrongness ratings as parametric modulators of the regressors of interest (i.e. ‘harm-to-other’ and ‘harm-to-self’). First-level contrast images were calculated by applying appropriate linear contrasts and were entered into second-level analyses.

Second-level one-sample t-tests were conducted for each contrast using the summary-statistics approach to random-effects analysis. Whole-brain analyses were conducted using a cluster-forming threshold of *p* < 0.001 (uncorrected), k > 10, and regions were reported as significant if they survived cluster-level whole-brain FWE correction (WBC) at p < 0.05. ROI analyses in the TPJ, SMG, aINS, amygdala and vmPFC were conducted using an initial threshold of p < 0.001 (uncorrected) and responses were considered significant if they survived voxel-level small volume FWE correction (SVC) at p < 0.05. ROIs were anatomically defined using masks from the automated anatomical labelling (AAL) atlas^39^ (see ‘Supplementary Experimental Procedures’ for full details). To identify associations between brain responses and psychopathic traits, each facet of the SRP was entered as a regressor of interest in these second level models.

To identify regions showing significant activation differences between the two tasks (guilt and wrongness ratings), and differences in associations between activation and psychopathic traits, two additional two-sample t-test models were specified. In the first model, the main effect contrasts of the two tasks were compared ([harm-to-other > harm-to-self in moral judgment task] > [harm-to-other > harm-to-self in guilt task]). Antisocial behaviour traits were entered as a covariate to assess whether associations with brain response differed significantly across the two tasks in the vmPFC and amygdala ROIs. In the second model, the two parametric modulators were compared ([parametric modulation of wrongness judgment] > [parametric modulation of guilt]). Interpersonal psychopathic traits were entered as a covariate to assess whether associations with the encoding of guilt differed significantly from the encoding of wrongness judgments in the aINS ROI. Age was added as a covariate in these analyses to control for the age difference between samples.

## Additional Information

**How to cite this article**: Seara-Cardoso, A. *et al.* Anticipation of guilt for everyday moral transgressions: The role of the anterior insula and the influence of interpersonal psychopathic traits. *Sci. Rep.* **6**, 36273; doi: 10.1038/srep36273 (2016).

**Publisher’s note:** Springer Nature remains neutral with regard to jurisdictional claims in published maps and institutional affiliations.

## Supplementary Material

Supplementary Information

## Figures and Tables

**Table 1:** Neural correlates of harm-to-others vs. harm-to-self scenarios.

Region	L/R	x	y	Z	Z	k	P_FWE_
**Guilt task**							
Ventromedial prefrontal cortex	L	−9	53	10	5.59	1288	<0.01
Supramarginal gyrus	R	54	−31	22	5.09	168	<0.01
Insula	R	39	5	1	4.89	337	<0.01
Postcentral gyrus	L	−54	−19	19	4.49	190	<0.01
Cerebellum	R	12	−64	−11	4.39	268	<0.01
Putamen	L	−27	−7	10	4.23	84	0.02
Ext. to insula	L	−36	2	16	4.01		
Posterior middle temporal gyrus	R	45	−67	10	4.01	100	0.01
Middle frontal gyrus	L	−27	44	28	3.86	94	0.01
Parahippocampal gyrus	L	−30	−13	−20	3.84	83	0.02
Amygdala^a^	R	30	−7	−17	4.23	9	<0.01
Amygdala^a^	L	−24	−7	−17	3.52	15	0.02
**Moral judgment task**							
Ventromedial prefrontal cortex	L	−9	56	7	5.98	1855	<0.01
Supramarginal gyrus	R	57	−16	28	5.15	114	<0.01
Cerebellum	R	21	−49	−26	4.83	89	0.01
Insula	R	36	5	7	4.47	475	<0.01
Insula	L	−45	2	4	4.42	481	<0.01
Amygdala^a^	R	24	2	−14	4.09	38	<0.01
Amygdala^a^	L	−15	−1	−17	3.36	2	0.03
Amygdala^a^	L	−27	−4	−14	3.28	4	0.03
**Guilt AND Moral Judgment tasks**							
Ventromedial prefrontal cortex	L	−9	56	7	6.06	1066	<0.01
Insula	R	39	5	4	4.65	146	<0.01
Supramarginal gyrus	R	57	−16	28	5.15	114	<0.01
Cerebellum	R	21	−49	−26	4.83	89	0.01
Insula	R	36	5	7	4.47	475	<0.01
Insula	L	−45	2	4	4.42	481	<0.01
Amygdala^a^	R	30	−7	−17	3.97	8	<0.01
Amygdala^a^	L	−27	—	−17	3.37	4	0.02

**Note:** Regions are reported at p < 0.05, whole-brain FWE cluster-corrected, after an initial threshold of P < 0.001 (uncorrected), k > 10, unless otherwise stated. Spatial coordinates (x, y, z) are in Montreal Neurological Institute space. R = Right; L = Left. ^a^Regions reported at P < 0.05, small-volume FWE voxel-level corrected within a bilateral anatomical amygdala mask, following an initial threshold of P < 0.001 (uncorrected).
