# Supplementary material for: Anticipation of guilt for everyday moral transgressions: The role of the anterior insula and the influence of interpersonal psychopathic traits
Source: Sci Rep. 2016 Nov 3;6:36273. doi: 10.1038/srep36273 (PMC5093734; doi:10.1038/srep36273)
Supplement: Supplementary Information [file srep36273-s1.pdf]

Anticipation of guilt for everyday moral transgressions: The role of the anterior insula and the influence of interpersonal psychopathic traits

Ana Seara-Cardoso[1,2]*, Catherine L. Sebastian[3], Eamon McCrory[1], Lucy Foulkes[1,4], Marine Buon[1], Jonathan P. Roiser[4]**, Essi Viding[1, 4]**

[1] Division of Psychology and Language Sciences, University College London, UK

[2] Neuropsychophysiology Lab, CiPsi, University of Minho, Portugal

[3] Department of Psychology, Royal Holloway University of London, UK

[4] Institute of Cognitive Neuroscience, University College London, UK

Supplementary Information

Supplementary Results

Table S1. Correlations between ratings of guilt and wrongness judgments in harm-to-other and harm-to-self scenarios

	**Total**	**Affective Traits**	**Interpersonal Traits**	**Lifestyle Traits**	**Antisocial Behaviour**
	R	r	r	r	r
**Guilt in HO**	-.52**	-.26	-.53**	.43*	-.24
**Guilt in HS**	-.29	-.15	-.27	-.24	-.22
**Wrongness Judgments in HO**	-.22	-.19	-.28	-.02	-.01
**Wrongness Judgments in HS**	-01	-04	-.01	-.00	.01

Notes: Pearson correlation coefficients are reported; * $P < 0.05$, ** $P < 0.01$ (2-tailed).

Table S2. Mean and variance differences in psychopathic traits and age between sample 1 (guilt task) and sample 2 (moral judgment task)

	**Levene's Test for Equality of Variances**		**t-test for Equality of Means**						
								**95% Confidence Interval of the Difference**	
	**F**	**Sig.**	**t**	**df**	**Sig. (2-tailed)**	**Mean Dif.**	**Std. Error Dif.**	**Lower**	**Upper**
**Total Traits**	2.31	0.13	0.65	54	0.52	2.18	3.33	-4.50	8.86
**Affective Traits**	0.04	0.85	-0.76	54	0.45	-0.75	0.99	-2.73	1.23
**Interpersonal Traits**	0.26	0.61	0.27	54	0.79	0.36	1.32	-2.29	3.00
**Lifestyle Traits**	2.88	0.10	0.84	54	0.41	1.14	1.37	-1.60	3.88
**Antisocial Behaviour**	1.34	0.25	1.83	54	0.07	1.36	0.74	-0.13	2.85
**Age**	5.86	0.02	2.62	54	0.01	3.25	1.24	0.77	5.73

Table S3. Descriptive statistics of psychopathic traits

	Mean	SD	Min	Max
Sample 1				
Total traits	54.68	14.01	31	80
Affective traits	12.93	3.55	7	19
Interpersonal traits	14.21	5.11	7	24
Lifestyle traits	16.11	6.14	7	31
Antisocial Behaviour	10.29	2.97	7	17
Sample 2				
Total traits	52.50	10.71	31	70
Affective traits	13.68	3.84	8	22
Interpersonal traits	13.86	4.77	7	22
Lifestyle traits	14.96	3.82	7	21
Antisocial Behaviour	8.93	2.58	7	17

Supplementary Experimental Procedures

Participants

In the first study (guilt version of the everyday moral transgressions task), one participant was excluded for excessive response failure (>50%) and MR signal artefact, one for excessive MR signal artefact, and two for being univariate outliers on the SRP questionnaire and multivariate outliers in SRP analyses. In the second study (wrongness judgment version), one participant was excluded due to an incidental MRI finding, one for excessive MRI signal artefact, and two for being univariate outliers on the SRP questionnaire and multivariate outliers in SRP analyses.

Magnetic resonance imaging acquisition

The T2* EPI sequence used the following acquisition parameters: 35 2 mm slices acquired in a descending trajectory with a 1 mm gap; echo time= 50 ms; repetition time=2975 ms; slice tilt=-30º (T > C); flip angle=90º; field of view=192 mm; matrix size=64 x 64. Functional data were acquired in a single run. Fieldmaps were also acquired for use in the unwarping stage of data preprocessing.

Image pre-processing and analysis

EPI data were analysed using SPM8 (www.fil.ion.ucl.ac.uk/spm). The first five volumes were discarded, and data were realigned to the sixth volume, unwarped using a fieldmap, normalized to the Montreal Neurological Institute template resampling to a voxel size of 2x2x2 mm, and smoothed with an 8 mm full width at half-maximum Gaussian filter. Data were high-pass filtered at 128 s to remove low-frequency drifts, and the statistical model included an AR(1) autoregressive function to account for autocorrelations.

For each version of the task (guilt and wrongness judgment), two first-level models were estimated for each participant, the first to identify brain regions that responded differentially to overall processing of moral transgressions (harm-to-self vs harm to-other scenarios), and the second to identify specific regions that parametrically encoded the strength of feelings of guilt, or of wrongness judgment, elicited by the moral transgressions. In the guilt version of the task, for one participant, an extra regressor was included in main-effect and parametric models to model 3 corrupted images resulting from excessive motion. These images were removed and the adjacent images interpolated in order to prevent distortion of the between-subjects mask.

Regions-of-interest

ROIs were anatomically defined using masks from the automated anatomical labelling (AAL) atlas The aINS ROI consisted of right and left insula, anterior to y = 0; the amygdala ROI consisted of right and left amygdala; the TPJ ROI included bilateral angular and superior temporal gyrus, posterior to y = -40, as described in Hutcherson et al. (2015); the SMG ROI included right and left SMG; and the vmPFC ROI included bilateral anterior cingulate cortex, rectu and medial orbitofrontal gyrus inferior to z = 0, also as defined in Hutcherson et al. (2015).

Task scenarios

ID		Setup	Outcome: Moral transgression (Harm-to-Other)	Outcome: Control (Harm-to-Self)
**1**	**Tube**	You are on a crowded tube on your way home. You are very tired. All you want to do is sit down and read your book. Fortunately you find an empty seat and sit down.	Suddenly you realise a heavily pregnant woman is standing by your side. You are so tired that you pretend not to see her so that you do not have to give up your seat.	Suddenly you realise you dropped your book by the door. Since you are so tired, you do not get up to fetch it in case you lose your seat to the person standing next to you.
**2**	**Manager**	You are hoping for a promotion in your job. During a meeting, your team manager praises the team's work. He is very complimentary about a novel idea the team has introduced.	He says the idea was yours. You know it was not; it was your colleague's. But you don't say anything because you don't want to risk your promotion.	He says the idea was your colleague's. You know it was your idea but you don't say anything because you don't want to risk your promotion.
**3**	**Station**	You are running late to catch a train to see your favourite band in concert. The station is very crowded and there is a group of tourists blocking the entrance to the platform.	They don't seem to be moving. To get through, you shove the person in front of you and he falls to the ground.	They don't seem to be moving. To get through, you try to go around the group but you trip on one of them and hurt your leg.
**4**	**Girlfriend**	You are on a holiday with your new girlfriend. There is a running competition on and you decide to enter it to impress her.	Another competitor looks to be in very good shape. To win the race you trip him up; he falls down and you win first place.	Another competitor looks to be in very good shape. You manage to overtake him, but you push yourself too hard and end up with a strained hamstring.
**5**	**Deadline**	It is late in the evening. You and your colleague are working late because you each have very important deadlines tonight.	You accidently spill your coffee and damage some keys on your keyboard. While your colleague takes a break you switch the keyboards so you won't risk missing the deadline.	You accidently spill your coffee and damage some keys on your keyboard. You have to wait until your colleague finishes his work to use his keyboard so you won't miss the deadline.
**6**	**Mobile**	Your mobile phone is broken. You need to buy a new one but you do not have that much money at the moment.	Walking out of your local café you see someone leaving a smartphone behind. The person seems to be very engaged talking to a friend so you quickly turn it off and keep it for yourself.	You buy a cheap replacement. Walking out of your local café you get distracted by someone else's conversation, drop the phone on the floor and break it. Now, you have to buy yet another phone.
**7**	**Noise**	It is late in the evening and you are preparing to go to bed. Suddenly, you hear a loud noise outside on the street.	You go to the window and see that someone is trying to steal your neighbour's car. You just want to go to bed so you ignore what's going on outside.	Your neighbour knocks on your door saying a tree branch has fallen on your car crashing the windscreen. You just want to go to bed so you don't do anything about it and leave it as it is.
**8**	**Parking**	You have been driving for a long time looking for a place to park your car in the supermarket car park.	You finally find an empty space but there is someone in a car waiting already and giving a signal to park. You ignore him and cut in front of him to take the space.	You finally find an empty space and park. You are about to leave the car when you realise you forgot your wallet at home and need to go back to get it.
**9**	**Car Insurance**	You need to save money to pay for your car insurance which has gone up unexpectedly. You arrive at your workplace and park at the same time as your colleague.	While parking, you accidentally ram the back of your colleague's car damaging it badly. You notice he looks confused and tell him it was his fault so you won't lose your savings paying for the damage.	When saying hello to your colleague you ram the front of your car into a pole, damaging it badly. Fixing the damage will cost you your no-claims bonus, which will make your insurance even more expensive.
**10**	**Queue**	You are at the supermarket getting food and drinks for your friends who are coming to your place later on in the afternoon.	There is a big queue at the tills. You lie and tell the person in front of you your grandmother is waiting in the car so that you jump the queue and don't have to wait.	There is a big queue in front of you. When there is finally only one person ahead of you, you realise you forgot the crisps. You go back to get them and lose your place.

11	**Dishes**	It is your turn to do the dishes. You don't feel like doing them and just want to go to the pub where your friends are.	You tell your housemate that you had an emergency call and need to go to your mother's place immediately. He offers to do the dishes for you, and you go out to the pub.	Your roommate reminds you that you forgot to buy washing up liquid. You need to go out and buy some. By the time you are done with the dishes your friends have left the pub.
12	**Night Out**	You are on a night out with a friend. You haven't been on a night out in a while and just want to have a good time.	Your friend is being a bore. You bump into a hilarious old acquaintance and just ignore your friend for the rest of the night.	You bump into an old acquaintance. He is very drunk and is a real bore but you can't get rid of him the whole evening.
13	**Football**	You are playing football with your friends and playing in defence. You really want to win. There is a guy on the other team who keeps passing you and scoring. Your team is getting behind.	You can't afford to let him score any more goals. Next time he approaches you, you tackle him badly injuring his leg, and he can't play the rest of the game.	Trying to stop him from scoring again you hurt your leg, but there is no-one else to take your place. You want your team to win so you have to keep playing while hurt.
14	**Induction**	You want to join a private club at university. You are a first year student and are going through the "induction". One of the senior students sets up a task.	You have to coerce another student to drink beer mixed with vodka until he throws up. You go along because you want to get into the club. The other student ends up passing out and needing his stomach pumped at the hospital.	You have to drink beer mixed with vodka until you throw up. You go along with this because you want to get into the club. You end up passing out and needing your stomach pumped at the hospital.
15	**Comp Game**	You have finally saved up enough money to buy a computer game that has just come out. You have just bought the game and can't wait to play.	Ages ago you promised your girlfriend that you would go out tonight, but you pretend you're ill so you can stay in and play the game.	Your girlfriend has let you have the evening to yourself to play. Only half an hour in, there is a powercut and you have to wait 3 hours before you can play again.
